# P01-007 – Evaluation of potential risk factors of Amyloidosis

**DOI:** 10.1186/1546-0096-11-S1-A11

**Published:** 2013-11-08

**Authors:** T Kasifoglu, S Bilge, E Gonullu, F Bekdemir, C Korkmaz

**Affiliations:** 1 Department Of Internal Medicine Division Of Rheumatology, Eskisehir Osmangazi University Medical Faculty, Eskisehir, Turkey

## Introduction

Familial Mediterranean Fever (FMF) is a genetic disease which is frequently seen in Middle East Region. The most common reason of morbidity and mortality is the end stage renal failure due to amyloidosis.

## Objectives

The potential risk factors of amyloidosis are known as ethnic origin (Jewish, Armenian, Turkish, and North African origin), non-usage of colchicines, and family history. Various investigators suggest that M694V mutation, especially homozygote pattern, is a risk factor for amyloidosis. In literatures reported from Turkey, it is stated that there is only a limited association. In this study, we aimed to investigate the affects of MEFV mutations and other potential risk factors on amyloidosis.

## Methods

The findings of 396 FMF patients of our clinic was retrospectively evaluated such as amyloidosis, MEFV mutations, gender, clinical features, family history, ages at initial symptoms, ages at diagnosis, the duration of colchicin usage. Amyloidosis was diagnosed by rectal or renal biopsies of patients with proteinuria over than 500 mg/day.

## Results

Renal amyloidosis was diagnosed at 8.3% (33) patients. There was no difference between patients with amyloidosis and without amyloidosis in terms of clinical features, family history, ages at initial symptoms, ages at diagnosis, the duration of colchicin usage. MEFV mutation was studied in 159 of 363 patients without amyloidosis and in 27 of 33 patients with amyloidosis. The mutations detected in patients with amyloidosis were M694V homozygote, M694V heterozygote, and compound heterozygote of M694V/V726A, M694V/M680I, V726A/M680I or V726A/R761H. The most common mutation was M694V homozygote of patients with and without amyloidosis (59.3% versus 32%). The frequency of M694V homozygote of patients with amyloidosis was statistically high from the frequency of patients without amyloidosis (p<0.001). But there was no difference in terms of other mutations.

## Conclusion

It is shown that, the only most common risk factor of Turkish patients is the M694V homozygote gene mutation in our study. But amyloidosis is not seen in all patients with M694V homozygote mutation. Therefore, large series are needed to evaluate the other potential risk factors. Close monitoring could be necessary for patients with M694V homozygote mutation.

## Disclosure of interest

None declared.

